# Bibliometric Analysis of Ebola Research Indexed in Web of Science and Scopus (2010-2020)

**DOI:** 10.1155/2020/5476567

**Published:** 2020-09-03

**Authors:** Joseph Kawuki, Xiaojin Yu, Taha Hussein Musa

**Affiliations:** ^1^Key Laboratory of Environmental Medicine Engineering, Ministry of Education, Department of Global Health, School of Public Health, Southeast University, Nanjing, 210009 Jiangsu Province, China; ^2^Key Laboratory of Environmental Medicine Engineering, Ministry of Education, Department of Epidemiology and Health Statistics, School of Public Health, Southeast University, Nanjing, 210009 Jiangsu Province, China; ^3^Biomedical Research Institute, Darfur College, Nyala, Sudan

## Abstract

**Background:**

Within the past decade, Africa has faced several recurrent outbreaks of Ebola virus disease (EVD), including the 2014-2016 outbreak in West Africa and the recent 2018-2020 Kivu outbreak in the Democratic Republic of Congo. The study thus aimed at quantifying and mapping the scientific output of EVD research published within 2010-2020 though a bibliometric perspective.

**Methods:**

EVD-related publications from 2010 to 2020 were retrieved from the Web of Science (WoS) and Scopus databases by using the keywords ‘Ebola', ‘Ebola Virus Disease', ‘Ebolas', and ‘ebolavirus'. Biblioshiny software (using R-studio cloud) was used to categorise and evaluate authors', countries' and journals' contribution. VOSviewer was used for network visualisation.

**Results:**

According to the used search strategy, a total of 3865 and 3848 EVD documents were published in WoS and Scopus, respectively. The average citation per document was 16.1 (WoS) and 16.3 (Scopus). The results show an overall increase in the publication trend within the study period. The leading countries in EVD research were the USA and UK, with over 100 papers in both databases, including Nigeria and South Africa. NIAID and CDC-USA were the most influential institutions, while “Infectious Diseases” and “Medicine” were the most decisive research fields. The most contributing authors included Feldmann H and Qiu XG with over 60 papers in each database, while *Journal of Infectious Diseases* was the most crucial journal. The most cited article was from Aylward et al. published in 2014, while recent years displayed a keyword focus on “double-blind”, “efficacy”, “ring vaccination” and “drug effect”.

**Conclusion:**

This bibliometric analysis provides an updated historical perspective of progress in EVD research and has highlighted the role played by various stakeholders. However, the contribution of African countries and institutions is not sufficiently reflected, implying a need for increased funding and focus on EVD research for effective prevention and control.

## 1. Introduction

Ebola virus disease (EVD) is a multifaceted zoonosis that is highly infectious in humans [1]. Ebola viruses that cause EVD are negative-stranded RNA viruses, belonging to the Filoviridae family and are endemic to regions of the west and equatorial Africa [2]. The first EVD human case was identified in 1976 in Zaire, the now called Democratic Republic of Cong-DRC [3]. Ebola virus is transmitted among humans via close and direct physical contact [4], and the animal-to-human transmission has been reported when people come into contact with tissues and bodily fluids of infected animals [3, 5].

Within the past ten years, Africa has faced several recurrent outbreaks of EVD, with case fatality rates, usually around 25–90% [1, 2]. Besides the 2014-2016 Ebola outbreak in West Africa, which claimed more than 1000 victims, Africa has been again stricken with the recent 2018-2020 Kivu Ebola outbreak in the DRC [6, 7]. This epidemic was caused by the Zaire ebolavirus species, which is the most lethal strain of the six known Ebola virus substrains [8]. On 17^th^ July 2019, World Health Organisation (WHO) announced the Kivu outbreak as a “Public Health Emergency of International Concern (PHEIC)” [9]. By 16^th^ December 2019, 3,348 cases and 2,210 deaths, including 161 healthcare workers, had been reported, with a fatality rate of 66% [6]. The overwhelming socioeconomic effects of this outbreak put the global health response in acute focus, with the potential of spreading to neighbouring countries [10].

The Government and the Ministry of Health (MoH) of DRC, WHO, and partners have tirelessly implemented several outbreak control interventions. However, the implementation of response measures remains a key challenge due to the prolonged humanitarian crisis in North Kivu province, the unstable security situation, and the mistrust of affected communities in response activities [7, 11]. Current evidence links the severity of the Ebola outbreak to the type of Ebola species involved. The Ebola Zaire and Ebola Sudan species are the most pathogenic, while Ebola Bundibugyo species appears to have a lower case fatality rate [2, 12].

Africa, within Ebola history, has witnessed serious outbreaks in the last decade, facilitating great research output. Besides, although EVD mainly affects Africa, it is a critical global health issue, which if not effectively controlled, could easily be exported all over the globe [13, 14]. This raises the need to collectively quantify and evaluate EVD research and give an updated historical perspective, owing to the considerable efforts and resources that have been injected into the control, treatment, and prevention of EVD. This study is thus aimed at mapping research efforts related to the Ebola virus disease published within 2010-2020 through a bibliometric perspective using documents indexed in the Web of Science and Scopus databases.

## 2. Materials and Methods

### 2.1. Study design

This study used a bibliometric analysis, a technic that has been progressively used as a tool and basis for monitoring research performance of various scientific disciplines, as well as supporting appropriate policy actions [15–17].

### 2.2. Data source

The study used the Web of Science Core Collection (WoS) [18] and Scopus databases. The selected databases cover most of the key international journals. Since the data used in this study were obtained from public databases and involved no direct interaction with human or animal subjects, ethical approval was not necessary.

### 2.3. Search strategy

The study used keywords: “Ebola”, “Ebola Virus Disease”, “Ebolas”, and “ebolavirus”, to retrieve EVD documents from WoS and Scopus published within 2010-2020 (6^th^ June). The keywords were searched in the article titles to maximise the accuracy of the retrieved research output. In order to include all published documents, the basic search method was used. From both databases, only English articles of categories “Original articles” and “Editorial material” were considered for analysis in this study.

### 2.4. Bibliometric Analysis

This study mainly reported descriptive statistics. The research trends and selected publication features from both databases were separately classified and analysed using “Biblioshiny app”—(using R-studio cloud) [19]—after which they were compared. These included the distribution of the most productive countries/territories, institutions, authors, research fields, journals, keywords, as well as *h*-index, impact factor, and total citations. In this regard, the journal impact factors were obtained from the “Journal Citation Reports (JCR)© Ranking: 2019” [20]. In addition, Microsoft Excel and VOSviewer (Van Eck & Waltman, Leiden University, The Netherlands) were used for data mining, mapping, and visualisation of the network analyses [21].

## 3. Results

**Figure 1:**
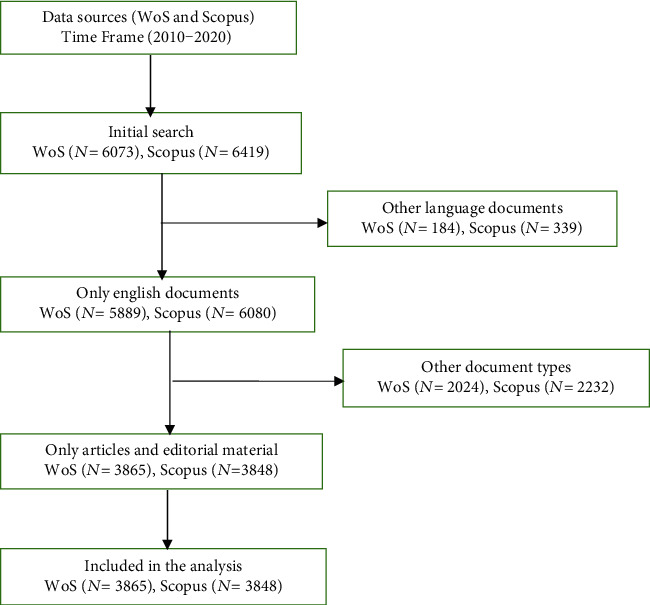
Flowchart of the methodology.

Using the search strategy in both WoS and Scopus: TITLE: (ebola) OR TITLE: (ebola virus disease) OR TITLE: (ebolas) OR TITLE: (ebolavirus), 6073 and 6419 documents were identified from WoS and Scopus, respectively (2010-2020). Of these, 96.9% (WoS) and 94.7% (Scopus) were English documents. After refining to only English language, articles, and editorial materials, 3865 and 3848 documents from WoS and Scopus, respectively, were included in the analysis, as shown in Figure 1.

### 3.1. Trends in Publication and Citation

Of the total publications from WoS, 3007 (77.8%) were research articles and 858 (22.2%) were editorial material, while from Scopus, 3562 (92.6%) were articles and 286 (7.4%) were editorial material.

**Figure 2:**
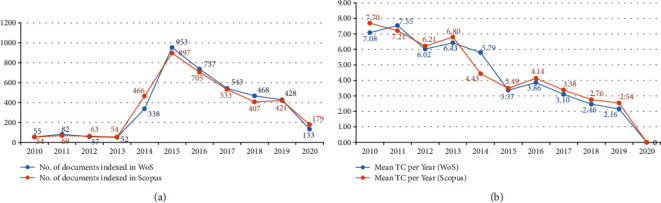
Annual trend of publication (a) and citation (b) from WoS and Scopus (2010-2020).

From 2010 to 2013, EVD publications from both databases were almost constant, with each less than 100 papers per year. However, the publications rapidly increased in 2014, attaining a maximum peak of over 800 publications from each database in 2015, after which they gradually reduced over the recent years (Figure 2(a)). The annual citation gradually decreased in both databases over the recent years, as shown in Figure 2(b). It should be noted that old documents tend to be cited more than newly published papers; hence, the observed decreasing trend in the citation score.

### 3.2. Most Contributing Countries and Their Collaborations

Authors' affiliations were analysed, which helped to identify the top prolific countries and institutions. Among the top 15 countries that contributed to EVD research output within the study period, the USA and the United Kingdom occupied the first two positions in both databases. In WoS, the USA and UK contributed to 1736 (44.9%) and 352 (9.1%), respectively, while in Scopus, they contributed 702 (18.3%) and 130 (3.4%) papers, respectively. Among the African countries, Nigeria was the top contributor with 52 (1.3%) papers in WoS and 17 (0.4) in Scopus.

Regarding single country papers (SCP), WoS had 488 (12.6%) SCP while Scopus had 758 (19.7%) SCP. The USA and UK still topped the list from both databases, as shown in Table 1.

**Figure 3:**
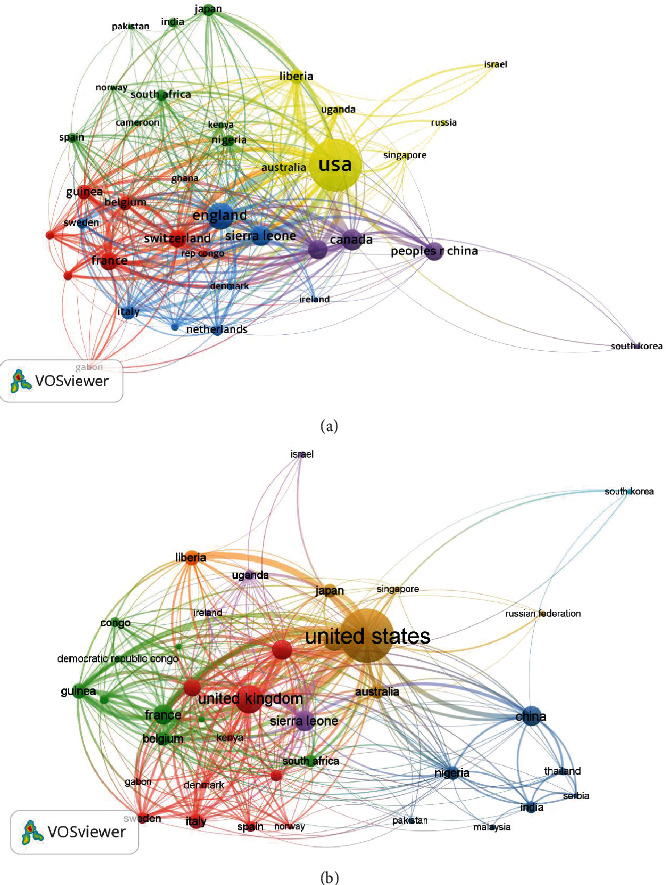
Network visualisation of collaborations among countries of EVD research indexed in WoS (a) and Scopus (b).

From both databases, the most crucial countries that showed collaborations with other countries in EVD research were the USA and UK. In WoS, the USA had 36 links (*L*), 1621 link strength (LS) and the UK had *L* = 34 and LS = 935, while in Scopus, the USA had *L* = 38 and LS = 1507 and UK had *L* = 35 and LS = 877. These were followed by Germany, Sierra Leone, Guinea, France, and South Africa, among others, as shown in Figure 3.

### 3.3. Most Productive Institutions and Their Collaborations

**Figure 4:**
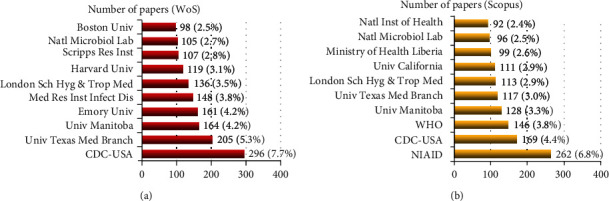
Ten most prolific organisations in EVD research in WoS and Scopus.

The most influential institutions from WoS were the Centre for Disease Control and Prevention, CDC-USA (296, 7.7%), University of Texas Medical Branch (205, 5.3%), and University of Manitoba (164, 4.2%). From Scopus, National Institute of Allergy Infectious Diseases-NIAID (262, 6.8%), CDC-USA (169, 4.4%), and World Health Organisation-WHO (146, 3.8%) topped the list (Figure 4).

**Figure 5:**
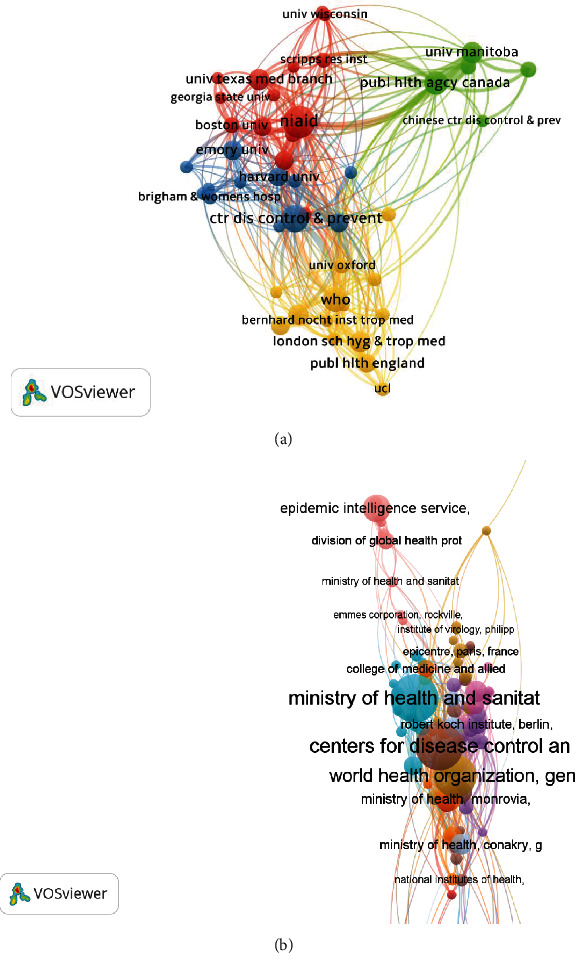
Network visualisation of collaborations among key institutions of EVD research from WoS (a) and Scopus (b).

Analysis of institutional collaboration revealed that NIAID (links, *L* = 39 and link strength, LS = 301), WHO (*L* = 36, LS = 239), and CDC-USA (*L* = 35, LS = 212) were the top three collaborative institutions of EVD research in WoS. While in Scopus, WHO (*L* = 42, LS = 83), Ministry of Health & Sanitation (*L* = 33, LS = 47), and CDC-USA (*L* = 29, LS = 61), among others were the most collaborative institutions as visualised in Figure 5.

### 3.4. Most Influential Funding Agencies and Research Fields

The results show that from both databases, US agencies were the dominant funders of EVD studies, and these included the United States Department of Health Human Services, National Institutes of Health USA, and National Institute of Allergy Infectious Diseases-NIAID, which all funded over 200 studies in both databases (Table 2**)**.

**Figure 6:**
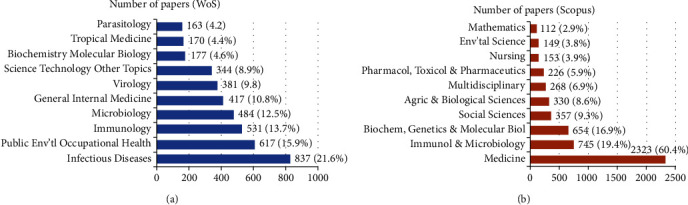
Most crucial research fields of EVD studies (2010-2020) in WoS and Scopus.

The top ten crucial fields in EVD research were categorised according to the WoS and Scopus fields and included “Infectious Diseases” (21.6%), “Public Environmental Occupational Health” (15.9%), and “Immunology” (13.7%), among others for WoS documents. On the other hand, “Medicine” (60.4%), “Immunology and Microbiology” (19.4%), and “Biochemistry, Genetics and Molecular Biology” (16.9%), among others were the most crucial research fields within the Scopus documents, as shown in Figure 6.

### 3.5. Most Contributing Authors and Their Collaborations

The analysed publications from WoS were produced by 15093 authors, of which 488 were authors of “single-authored documents,” while Scopus documents were from 15428 authors, of which 527 were authors of “single-authored documents.” The top three productive authors of EVD research within the study period included Feldmann H, Qiu XG, and Becker S, each with over 50 papers and gained over 2000 total citations in both databases, as shown Table 3.

The collaboration Iindex (CI) of documents from WoS was 4.55, with an average of 3.92 authors per document while 4.82 CI for Scopus documents, with an average of 4.01 authors per document.

**Figure 7:**
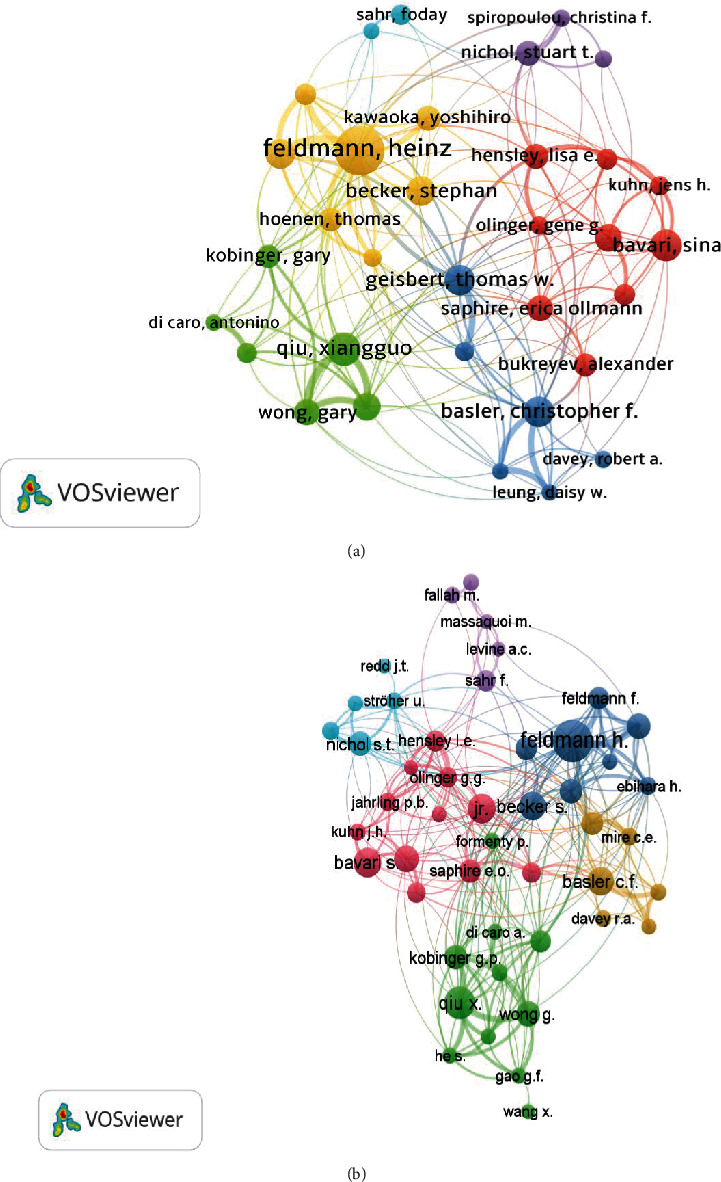
Coauthorship analysis of the most influential authors of EVD research (2010-2020) from WoS (a) and Scopus (b).

The most crucial collaborations among authors of EVD research in the retrieved WoS literature were that of Feldmann Heinz (links (*L*) = 20, link strength (LS) = 139), Geisbert TW (*L* = 18, LS = 74), and Marzi Andrea (*L* = 10, LS = 79), among others. For Scopus documents, Feldmann H (*L* = 23, LS = 147), Qui XG (*L* = 17, LS = 118), and Basler CF (*L* = 14, LS = 68), among others were the most collaborating authors (Figure 7).

### 3.6. Most Productive Journals

From WoS, the analysed documents were published by 943 journals while those from Scopus were produced by 1143 journals. The ten most productive journals from both databases are listed below, where *Journal of Infectious Diseases* and *Journal of Virology* topped the lists of both databases (Table 4).

### 3.7. Most Cited Documents

The analysed documents from WoS had a mean citation of 16.13 per document, while those from Scopus had 16.33 citations per document. The most cited article in both databases belongs to Aylward et al. and was published in *The New England Journal of Medicine* in 2014, under the title “Ebola Virus Disease in West Africa-the First 9 Months of the Epidemic and Forward Projections”, with 830 and 940 total citations (TC) in WoS and Scopus, respectively. This was followed by a brief report of Baize et al. and other research articles, as shown in Table 5.

### 3.8. Keyword Analysis

**Figure 8:**
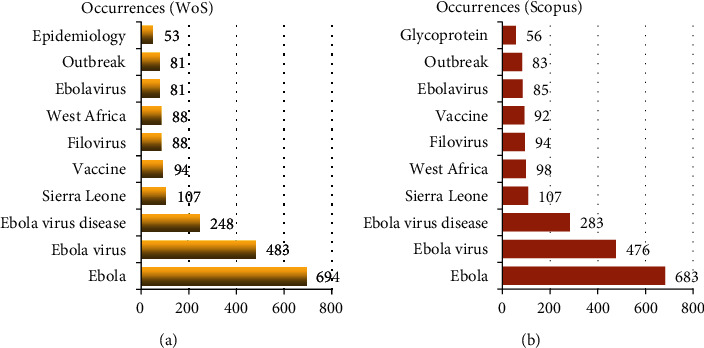
Most ten frequent author keywords (2010-2020) in WoS and Scopus.

**Figure 9:**
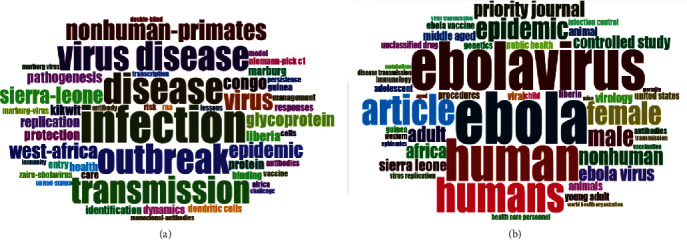
Visualised word-clouds of keywords plus (2010-2020) in WoS (a) and Scopus (b).

On analysis, WoS documents had 3857 keywords plus and 4369 author keywords, while Scopus documents had 11553 keywords plus and 4387 author keywords. Keyword distribution was analysed to detect directions and topics in EVD research and to understand discipline development. The most frequent author keywords were “Ebola,” “Ebola virus,” and “Ebola virus disease” with over 200 occurrences in both databases, as shown in Figure 8. Besides, the most frequent keywords plus included “infection”, “Ebola”, and “outbreak”, as visualised in Figure 9. The size and centrality of the word reflect its frequency and magnitude. Note that keywords plus are words that frequently appear in the titles of article's references, but do not appear in the title of the article itself, and are vital in exploring the knowledge structure of scientific fields.

Trends of topics basing on the frequency of keywords plus indicated that, in 2018, topics with keywords such as “double-blind”, “efficacy”, and “immunogenicity” dominated in WoS, followed by topics with “ring vaccination”, “candidate”, and “adults” in 2019. For Scopus documents, topics with keywords like “DRC”, “drug effect”, and “cohort analysis” dominated in 2018, followed by topics of “veterinary medicine”, “whole genome sequencing”, and “coronavirus infection”, among others in 2019-2020, as shown in Supplementary file 1.

## 4. Discussion

This bibliometric analysis presented an updated insight in the trend and scientific output of EVD-related publications. The initial search revealed that over 90% of the publications from both databases were in English, implying that English is the broadest language used in most official and international communications. Besides, research articles and editorial material were included in this study because, in times of disease outbreaks and emergencies, the first findings or scientific communications are usually published in the form of editorials. Nevertheless, these two types of documents contributed to over 60% of the EVD publications in both databases, which directly translates into the efforts rendered towards EVD research during the study period.

Trends in EVD publication output showed a rapid increase from 2014 with a peak in 2015. This was a period of the 2014-2016 Ebola outbreak in West Africa, the largest outbreak in EVD history that spread to several countries in West Africa, claiming several thousands of lives [22]. The analysis also identified a significant publication output from both databases between 2018 and 2020, which is as a result of the recent 2018-2020 Kivu EVD outbreak in the DRC [6]. The research output is expected to increase throughout 2020 owing to the 11^th^ EVD outbreak declared on 1^st^ June 2020 [23], and the newly, plus the yet to be, approved Ebola vaccines [24].

Among the top countries contributing to EVD research, the USA and the UK, among other Western countries, dominated the lists in both databases. Only, Nigeria and South Africa appeared in the top ten productive countries. The results align with Yi et al. [25] and Pouris and Ho [26], implying that the USA has maintained its leading role in not only EVD research but also other research fields. Besides, the top influential institutions included NIAID, CDC-USA, and WHO, among others, with only one African institution on the list of the top ten. Given the fact that EVD is endemic and mostly affects African countries [27], the dominancy of Western countries and institutions in EVD research implies a need for more focus and involvement of African countries in EVD research.

Network analysis showed that the WHO, CDC, and NIAID exhibited the highest degree of collaboration within EVD research. Such institutions operate within various countries, especially in Africa, where they support the Ministries of Health, thus explaining their dominant institutional partnerships. Besides, the analysis showed the most evident country collaborations to be that of the USA, UK, and France. Several African countries also exhibited a significant degree of cooperation. In one perspective, this may demonstrate the Public Health Diplomacy (PHD) exhibited by the international community and the role of partnerships in managing disease outbreaks and global emergencies. However, since African countries mainly contributed in the form of collaborations (with less single-country papers), it implies a need for African countries to develop and improve their research facilities to enable then carry out independent research.

With regard to funding EVD research, mostly the US and Western agencies topped the list, with no African agency among the top ten. This implies a need for more funding of EVD research from African governments and institutions. The most key EVD research fields were “Infectious Diseases” and “Medicine,” among others. This could imply the top fields that attract funds within EVD research, as well as the key funding institutions of such fields.

Besides, the analysis noted that the top journals of EVD research are foreign journals, which included *Journal of Infectious Diseases* and *Journal of Virology*, among others. This implies that most of the valuable EVD research findings and recommendations are published in foreign journals, whose accessibility by the African community is questionable. These recommendations may just remain on papers unutilised if they are not freely or easily accessible. Therefore, there is a need for African countries to improve and strengthen local research databases and journals for easier dissemination of EVD research findings.

The study results identified the most contributing authors of EVD research, which included Feldmann H, Qiu XG, and Becker S, among others. Furthermore, network analysis revealed authors with the highest collaborations. This information would be helpful to future researchers in this field, to quickly identify the crucial possible researchers for partnership or even consultation.

The most highly cited article was written by Aylward et al. in 2014; this was the first document to give a general insight into the 2014-2016 EVD outbreak, which had already spread to five countries by then. The paper tried to account for the first 9 months of the epidemic and predicted a rise in the number of cases from hundreds to thousands if no effective control measures implemented [22].

Keyword analysis revealed the most common keywords plus, which included “hemorrhagic-fever” and “Ebola”, among others. The dominancy of topics with keywords like “double-blind”, “efficacy”, “immunogenicity” “ring vaccination”, and “DRC”, among others in 2018 reflects the efforts put in formulating and testing new effective Ebola vaccines in the Kivu outbreak [28]. Besides, the dominancy of topics of “veterinary medicine” and “coronavirus infection” suggests a link between EVD and COVID-19, as both are zoonotic diseases which can be transmitted by bats to the human population and have similar prevention and control measures [4, 29].

Unlike other previous similar bibliometric studies, this one concurrently analysed and compared two databases (WoS and Scopus). However, this study also had some limitations, like the analysis underestimated publications in other languages, although few, they could be of value in this field.

## 5. Conclusions

The current bibliometric analysis summarised EVD research output (2010-2020), in which it highlighted an enormous publication output during the study period. The study has identified the leading roles played by various stakeholders in addressing EVD, in which it noted the top productive countries, authors, institutions, and journals. However, the contribution of African countries is not sufficiently reflected, thus implying a need for more focus, involvement, and funding of EVD research for effective prevention and control of EVD. Besides, the study noted a significant degree of collaboration among various stakeholders. This is vital, as it enables knowledge sharing and transfer also required for effective prevention and control, as well as vaccine development.

## Figures and Tables

**Table 1:** Top 15 productive countries of EVD research (2010-2020).

Rank	WoS			Scopus		
	Country	Number of papers (%), *N* = 3865	Single country papers	Country	Number of papers (%), *N* = 3848	Single country papers
1	USA	1736 (44.9)	1189	USA	702 (18.3)	479
2	United Kingdom	352 (9.1)	180	United Kingdom	130 (3.4)	66
3	China	198 (5.1)	126	Canada	76 (2.0)	37
4	Canada	160 (4.1)	80	France	65 (1.7)	26
5	Germany	134 (3.5)	62	Germany	65 (1.7)	29
6	France	122 (3.2)	52	China	57 (1.5)	33
7	Australia	75 (1.9)	42	Italy	39 (1.0)	21
8	Switzerland	75 (1.9)	30	Japan	34 (0.9)	17
9	Italy	62 (1.6)	32	Switzerland	31 (0.8)	8
10	Japan	57 (1.5)	25	Georgia	23 (0.6)	14
11	Nigeria	52 (1.3)	30	India	23 (0.6)	21
12	Belgium	46 (1.2)	9	Spain	20 (0.5)	12
13	South Africa	42 (1.1)	18	Netherlands	19 (0.5)	10
14	India	41 (1.1)	36	Nigeria	17 (0.4)	10
15	Spain	41 (1.1)	23	Australia	16 (0.4)	6

**Table 2:** Top 10 funding agencies of EVD research (2010-2020).

Rank	Funding agencies (WoS)	No. of papers (%), *N* = 3865	Funding agencies (Scopus)	No. of papers (%), *N* = 3848
1^st^	US Dept. of Health Human Services	742 (19.2)	National Institutes of Health USA	422 (10.9)
2^nd^	National Institutes of Health USA	691 (17.9)	NIAID	205 (5.3)
3^rd^	NIAID	303 (7.8)	Defense Threat Reduction Agency	90 (2.3)
4^th^	US Depart. of Defense	160 (4.1)	National Science Foundation NSF	82 (2.1)
5^th^	Defense Threat Reduction Agency	124 (3.2)	National Natural Science Foundation of China	78 (2.0)
6^th^	Wellcome Trust	106 (2.7)	Centers for Disease Control and Prevention	59 (1.5)
7^th^	National Natural Science Foundation of China	100 (2.6)	Wellcome Trust	54 (1.4)
8^th^	World Health Organization	97 (2.5)	Deutsche Forschungsgemeinschaft	45 (1.2)
9^th^	National Science Foundation NSF	95 (2.5)	World Health Organization	45 (1.2)
10^th^	European Union EU	84 (2.2)	National Institute of General Medical Sciences	44 (1.1)

**Table 3:** Top 10 productive authors of EVD research (2010-2020).

Rank	WoS				Scopus			
	Author	*h*-index	Total citations	No. of papers	Author	*h*-index	Total citations	No. of papers
1^st^	Feldmann H	35	3851	96	Feldmann H	33	3147	80
2^nd^	Qiu XG	26	2396	66	Qiu X	26	2564	63
3^th^	Becker S	22	2211	53	Becker S	23	2331	51
4^th^	Bavari S	22	1312	52	Bavari S	21	1361	49
5^th^	Nichol ST	28	2375	51	Nichol ST	26	2462	49
6^th^	Geisbert TW	24	2736	50	Dye JM	24	1997	44
7^th^	Kobinger GP	25	2232	49	Basler CF	25	1711	42
8^th^	Marzi A	23	1517	48	Marzi A	23	1458	42
9^th^	Saphire EO	21	1386	48	Kobinger GP	25	2409	41
10^th^	Dye JM	24	1916	46	Wong G	19	1848	40

**Table 4:** Top 10 journals of EVD research.

Rank	WoS			Scopus		
	Journal name	No. of papers (%), *N* = 3865	Impact factor (2019)	Journal name	No. of papers (%), *N* = 3848	Impact factor (2019)
1^st^	Journal of Infectious Diseases	223 (5.8)	4.73	Journal of Infectious Diseases	146 (3.8)	4.73
2^nd^	Journal of Virology	107 (2.8)	4.16	Journal of Virology	107 (2.8)	4.16
3^rd^	Lancet	105 (2.7)	43.38	PLoS One	100 (2.6)	2.87
4^th^	PLoS One	101 (2.6)	2.87	PLoS Neglected Tropical Diseases	91 (2.4)	4.40
5^th^	Emerging Infectious Diseases	86 (2.2)	6.81	Emerging Infectious Diseases	82 (2.1)	6.81
6^th^	Morbidity and Mortality Weekly Report	85 (2.2)	14.40	Scientific Reports	72 (1.9)	4.12
7^th^	PLoS Neglected Tropical Diseases	83 (2.1)	4.40	New England Journal of Medicine	52 (1.4)	37.91
8^th^	Lancet Infectious Diseases	82 (2.1)	21.77	Morbidity and Mortality Weekly Report	51 (1.3)	14.40
9^th^	Scientific Reports	72 (1.9)	4.12	Viruses	49 (1.3)	3.76
10^th^	New England Journal of Medicine	60 (1.5)	37.91	Antiviral Research	42 (1.1)	4.13

**Table 5:** The top 10 most cited EVD documents in WoS and Scopus (2010-2020).

Authors, year	Document title and journal name	Document type	TC (WoS)	TC (Scopus)
Aylward et al. 2014	Ebola virus disease in West Africa-the first 9 months of the epidemic and forward projections, The New England Journal of Medicine	Research article	830	940
Baise et al. 2014	Emergence of Zaire Ebola virus disease in Guinea, New England Journal of Medicine	Brief report	753	871
Feldmann Geisbert 2011	Ebola haemorrhagic fever, The Lancet	Research article	700	-N/A-
Gire et al. 2014	Genomic surveillance elucidates Ebola virus origin and transmission during the 2014 outbreak, Science	Research article	635	685
Carette et al. 2011	Ebola virus entry requires the cholesterol transporter Niemann–Pick C1, Nature	Research article	630	662
Qiu et al. 2014	Reversion of advanced Ebola virus disease in nonhuman primates with ZMapp, Nature	Research article	557	613
Henao-Restrepo et al. 2015	Efficacy and effectiveness of an rVSV-vectored vaccine expressing Ebola surface glycoprotein: interim results from the Guinea ring vaccination cluster-randomised trial, The Lancet	Research article	441	473
Quick et al. 2016	Real-time, portable genome sequencing for Ebola surveillance, Nature	Research article	430	459
Côté et al. 2011	Small molecule inhibitors reveal Niemann–Pick C1 is essential for Ebola virus infection, Nature	Research article	379	399
Schieffelin et al. 2014	Clinical illness and outcomes in patients with Ebola in Sierra Leone, New England Journal of Medicine.	Research article	332	366

## Data Availability

All research data used to support the findings of this study are included within the article, and the analysed data used can be freely accessed from the Web of Science data collection and Scopus.

## Supplementary Materials

Supplementary Materials (a) Showing the trend of topics based on keywords plus from Web of Science (2010-2020) and (b) showing the trend of topics based on keywords plus from Scopus (2010-2020).
