# Supplementary material for: Bibliometric Analysis of Ebola Research Indexed in Web of Science and Scopus (2010-2020)
Source: Biomed Res Int. 2020 Sep 3;2020:5476567. doi: 10.1155/2020/5476567 (PMC7486633; doi:10.1155/2020/5476567)
Supplement: Supplementary Materials — (a) Showing the trend of topics based on keywords plus from Web of Science (2010-2020) and (b) showing the trend of topics based on keywords plus from Scopus (2010-2020). [file 5476567.f1.doc]

**Supplementary file 1:**


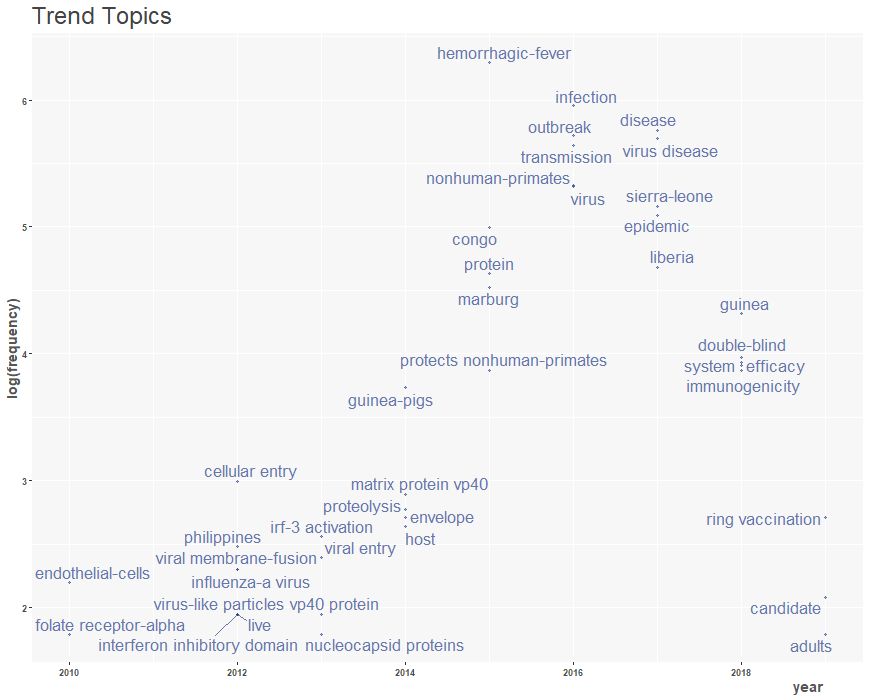


(a)

**The trend of topics basing on keywords plus from Web of Science (2010-2020)**


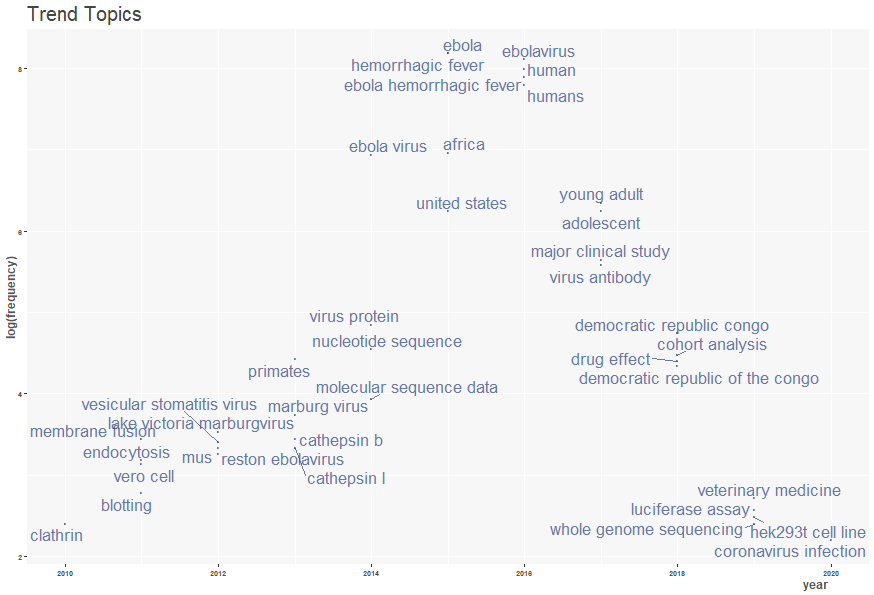


**The trend of topics basing on keywords plus from Scopus (2010-2020)**
